# Methylphenidate for treating tobacco dependence in non-attention deficit hyperactivity disorder smokers: A pilot randomized placebo-controlled trial

**DOI:** 10.1186/1477-5751-10-1

**Published:** 2011-01-28

**Authors:** Richard D Hurt, Jon O Ebbert, Ivana T Croghan, Darrell R Schroeder, Amit Sood, J Taylor Hays

**Affiliations:** 1 Nicotine Dependence Center, Mayo Clinic, 200 1st Street SW, Rochester, MN 55905 USA; 2 Primary Care Internal Medicine, Mayo Clinic, 200 1st Street SW, Rochester, MN 55905 USA; 3 Biomedical Statistics, Mayo Clinic, 200 1st Street SW, Rochester, MN 55905 USA; 4 General Internal Medicine, Mayo Clinic, 200 1st Street SW, Rochester, MN 55905 USA

## Abstract

**Background:**

Methylphenidate blocks the re-uptake of dopamine by binding to the dopamine transporter in the presynaptic cell membrane and increases extracellular dopamine levels. Similarities in neuropsychologic effects between nicotine and methylphenidate make it an intriguing potential therapeutic option. Previous research of methylphenidate in smokers has suggested a possible beneficial effect for the relief of nicotine withdrawal symptoms, but showed no efficacy in helping smokers with attention deficit hyperactivity disorder (ADHD) to stop smoking.

**Methods:**

To investigate potential efficacy for relieving nicotine withdrawal symptoms and promoting smoking abstinence, we conducted a randomized, double-blind, placebo-controlled, phase II study of once-a-day osmotic-release oral system methylphenidate (OROS-MPH, Concerta^®^) at a target dose of 54-mg/day for 8 weeks compared with placebo in 80 adult cigarette smokers.

**Results:**

Of the 80 randomized subjects and median smoking rate was 20 cigarettes per day. At the end of the medication phase, the biochemically confirmed 7-day point prevalence smoking abstinence was 10% (4/40) for the placebo group and 2.5% (1/40) for the OROS-MPH group.

Nicotine withdrawal was not found to differ significantly between treatment groups during the first 14 days following the start of medication prior to the target quit date (p = 0.464) or during the first 14 days following the target quit date (p = 0.786).

**Conclusion:**

We observed no evidence of efficacy of OROS-MPH to aid smokers to stop smoking. Although there are biologically plausible hypotheses that support the use of OROS-MPH for treating tobacco dependence, we found no evidence to support such hypotheses. In addition to no increase in smoking abstinence, we saw no effect of OROS-MPH for tobacco withdrawal symptom relief and no change in smoking rates was observed in the OROS-MPH group compared to the placebo group.

## Introduction

Expansion of pharmacologic options for treating tobacco dependence is needed. A large part of the positive reinforcement from cigarettes is due to the delivery of nicotine to the central nervous system (CNS), resulting in increased concentrations of dopamine in the reward centers of the brain [1]. Methylphenidate was considered as a potential treatment for smokers because of its action to block the re-uptake of dopamine by binding to the dopamine transporter in the presynaptic cell membrane and increase extracellular dopamine levels [2,3]. Similarities in neuropsychologic effects between nicotine and methylphenidate have also made methylphenidate an intriguing potential therapeutic option for tobacco dependence treatment [4]. Conversely, in laboratory studies, methylphenidate has been shown to increase smoking in adult non-ADHD smokers who were not trying to stop smoking [5,6]. To date, the only large trial of methylphenidate in smokers has been in smokers with Attention Deficit Hyperactivity Disorder (ADHD) who were all provided nicotine patch therapy and osmotic-release oral system methylphenidate (OROS-MPH, Concerta^®^) or placebo methylphenidate at a dose titrated to 72 mg/d. No difference was observed in the prolonged smoking abstinence between the two groups (43% vs. 42%) [7].

In order to explore the potential of methylphenidate to treat tobacco dependence, we conducted a randomized, double-blind, placebo-controlled, phase II study of OROS-MPH at a target dose of 54-mg/day for 8 weeks compared with placebo in 80 adult cigarette smokers without ADHD.

## Methods

The Mayo Foundation Institutional Review Board reviewed and approved the study protocol. Interested participants were recruited to the Mayo Clinic Nicotine Research Program from Rochester, MN and the surrounding area through news releases and advertisements. Subjects were eligible for enrollment if they were age 18 to 65 years, smoked ≥ 10 cigarettes for the past 6 months, were willing to make an attempt to stop smoking, and were able to provide written informed consent.

Exclusion criteria included: current major depressive or anxiety disorder; life-time diagnosis of bipolar disorder; schizophrenia or dementia; moderate to severe depression as determined by the Center for Epidemiologic Studies Depression Scale (CES-D); currently (in previous 30 days) using any tobacco dependence treatment program; have used an investigational drug within the past 30 days; history of alcohol or drug abuse or dependence as assessed using the CAGE questionnaire and the Drug Abuse Screening Test 20 (DAST-20); pregnant, lactating, or likely to become pregnant during the medication phase and not willing to use contraception; history of any major cardiovascular event in the past 6 months; an ECG with significant arrhythmias or abnormal conduction; currently taking medications known to interact with methylphenidate and not able to stop the medication during the study period; uncontrolled hypertension (> 160/100) or tachycardia (heart rate > 110); another household member participating in the study; known allergy to methylphenidate or its constituents; and a specific medical condition in which use of methylphenidate is contraindicated.

### Procedures

The study included a telephone pre-screen, 11 clinic visits, and one telephone follow-up visit. The clinic visits included an information meeting, an enrollment visit, 8 weekly visits during the medication phase, a telephone follow-up visit at 4 months, and an end-of-study clinic visit at 6 months. Qualified participants were informed of study procedures and other consenting issues, signed informed consent, then they completed screening questionnaires and interviews. All female study participants of childbearing age were required to have a negative pregnancy test at least 7 days prior to study drug initiation and agree to use approved contraception during study participation. Subjects returned for a baseline visit, completed additional screening questionnaires, underwent a medical history and physical examination by a study physician, and had a 12 lead electrocardiogram. Subjects were randomly assigned to placebo or active OROS-MPH at a dose of one 18 mg tablet a day with a dose escalation schedule in the first 2 weeks to achieve a maximum dose of 54 mg (three-18 mg tablets once daily). The target quit date (TQD) was set for day 14 after starting the study medication as it takes about 10 days to achieve the target dose of OROS-MPH. Participants reporting intolerable side effects were advised to go to the next lower dose.

The medication phase lasted for 8 weeks where subjects returned weekly for assessment of medication adherence and adverse events. Brief behavioral counseling (< 10 minutes) by a trained study assistant was completed at each visit using the "Smoke Free and Living It" manual. This manual, which allows for an individualized intervention, was designed by and has been utilized by the staff of the Nicotine Research Program for several years. Subjects received $10 for each completed clinic visit and $5 for each completed telephone visit.

### Assessments

At baseline, we assessed tobacco dependence utilizing the Fagerström Test for Nicotine Dependence [8], alcoholism using the CAGE questionnaire [9], substance dependence using the DAST-20 [10], and readiness to quit using the contemplation ladder [11]. We also assessed for depression using the Center for Epidemiologic Studies for Depression Scale (CES-D) [12]. The CES-D was repeated at week 4 and at the end-of-medication phase (week 8). Information on adverse events and concomitant medication was collected at each visit. Nicotine withdrawal and craving were assessed with a daily diary which contained the Minnesota Nicotine Withdrawal Scale (MNWS) and self-reported tobacco use [13]. Daily nicotine withdrawal data were obtained from the information meeting to week 5.

Medication adherence was assessed by conducting pill counts at each visit and by self reports about missed doses. Expired air carbon monoxide (CO) measured in parts per million (ppm) was obtained at every visit.

### Smoking Abstinence Outcomes

The primary endpoints were 7-day point prevalence and prolonged smoking abstinence at end of treatment (8 weeks). Secondary endpoints were 7-day point prevalence and prolonged smoking abstinence at 6 months. Smoking abstinence outcomes were analyzed using an intention-to-treat approach and subjects reporting use of tobacco products other than cigarettes were considered to be treatment failures. Point prevalence smoking abstinence was adjudicated by a negative response to the question, "Have you used any type of tobacco, even a puff, in the past 7 days?" and b) expired air CO ≤ 8 ppm [13] Prolonged smoking abstinence was adjudicated if criteria for 7-day point prevalence for smoking abstinence were satisfied and a negative response at each visit was obtained to the question, "Since (month/day/year) which was 2 weeks after your target-quit-date, have you smoked any tobacco, even a puff, for 7 consecutive days or at least once each week on 2 consecutive weeks?"

### Statistical Methods

Smoking abstinence at week 8 (end-of-medication) and week 24 was compared between groups using Fisher's exact test. Nicotine withdrawal symptoms and cravings were assessed daily using the MNWS. A composite withdrawal score was computed as the mean of the individual symptoms with craving analyzed separately. For each subject, the average score for the 7 days prior to the start of medication was used as baseline. Daily scores for the first 24 days following the start of medication were expressed as change from baseline and analyzed using generalized estimating equations (GEE). Since the target quit-date was the 15^th ^day following the start of medication, separate analyses were performed using data from days 1-14 and 15-28. In all cases,

the explanatory variables included in the models were treatment group (OROS-MPH vs. placebo) and time in days following start of medication. Daily withdrawal scores were also compared between groups using the two-sample t-test. Adverse events judged to be possibly, probably, or definitely related to study drug were summarized and compared between groups using Fisher's exact test.

## Results Subject characteristics

A total of 80 cigarette smokers were enrolled. Baseline subject characteristics were similar in the two treatment groups (Table 1). Of the 80 subjects randomized, there were 34 (16 placebo, 18 OROS-MPH) who discontinued study participation prior to the end of the medication phase. The reasons for discontinuation included: withdrawn consent (10 placebo, 2 OROS-MPH), lost to follow-up (4 placebo, 7 OROS-MPH), scheduling difficulty (2 placebo, 4 OROS-MPH) and adverse event (0 placebo, 5 OROS-MPH). Nearly all (87.5% placebo, 94.4% OROS-MPH) of those who discontinued the study during the medication phase reported smoking at the last study visit they attended.

**Table 1:** Baseline Characteristics

	Placebo	OROS-MPH
Characteristic	N = 40	N = 40
Age		
mean ± SD	38.0 ± 11.9	35.6 ± 11.0
median (range)	38 (20 to 69)	34 (19 to 57)
Gender, n (%)		
Male	14 (35)	20 (50)
Female	26 (65)	20 (50)
Race, n (%)		
White, non-Hispanic	38 (95)	37 (92)
Other*	2 (5)	3 (8)
Marital Status, n (%)		
Never married	8 (20)	13 (32)
Separated/divorced	11 (28)	11 (28)
Married/living as married	20 (50)	14 (40)
Widowed	1 (2)	0 (0)
Level of education, n (%)		
High school graduate or less	9 (22)	12 (30)
Some college or technical school	24 (60)	25 (62)
4-year college degree or more	7 (18)	3 (8)
Cigarettes per day		
mean ± SD	20.8 ± 8.2	19.7 ± 7.4
median (range)	20 (10, 45)	20 (10, 40)
Number of years smoked		
mean ± SD	18.8 ± 10.5	19.0 ± 10.4
median (range)	18 (3, 40)	20 (3, 40)
Fagerström Test for Nicotine Dependence		
mean ± SD	5.8 ± 1.8	5.2 ± 2.2
median (range)	6 (2, 10)	6 (1, 9)
Contemplation ladder		
mean ± SD	8.5 ± 1.3	8.8 ± 1.4
median (range)	9 (6, 10)	9 (6, 10)
Previous stop attempts, n (%)		
None	6 (15)	6 (15)
1 to 2	17 (42)	15 (38)
3 to 5	11 (28)	13 (32)
6 or more	6 (15)	6 (15)
Other tobacco users in the household, n (%)		
No	27 (68)	26 (65)
Yes	13 (32)	14 (35)

* These include: American Indian/Alaska native (n = 1), Asian (n = 1), Black (n = 2) and White Hispanic (n = 1).

### Smoking Abstinence and Smoking Reduction Outcomes

Smoking abstinence outcomes are presented in Table 2. At the end of the medication phase, the biochemically confirmed 7-day point prevalence smoking abstinence was 10% (4/40) for the placebo group and 2.5% (1/40) for the OROS-MPH group. Under the assumption that subjects who discontinue study participation were smoking at their baseline rate, the average number of cigarettes smoked per day in those who continued to smoke at the end of the medication phase was significantly (p < 0.001) lower than baseline for both treatment groups. However, the change from baseline did not differ significantly between treatment groups (-4.2 ± 7.5 and -5.6 ± 8.7 cigarettes per day for placebo and OROS-MPH respectively; p = 0.436). At week 24, the biochemically confirmed 7-day point prevalence smoking abstinence was 7.5% for the placebo group and 10% for the OROS-MPH group. In each group, only 1 subject (2.5%) met criteria for prolonged smoking abstinence through week 24.

**Table 2:** Smoking Abstinence Outcomes

	Placebo (N = 40)	OROS-MPH (N = 40)	
Week	No. (%)	No. (%)	p†
Week 12 (end of medication)			
7-day point prevalence*	4 (10.0)	1 (2.5)	0.973
Prolonged abstinence	4 (10.0)	1 (2.5)	0.973
Week 24			
7-day point prevalence*	3 (7.5)	4 (10.0)	0.500
Prolonged abstinence	1 (2.5)	1 (2.5)	0.753

* Biochemically confirmed by expired CO ≤ 8 ppm.

† One-tailed Fisher's exact test comparing OROS-MPH vs placebo.

### Nicotine Withdrawal

**Figure 1:**
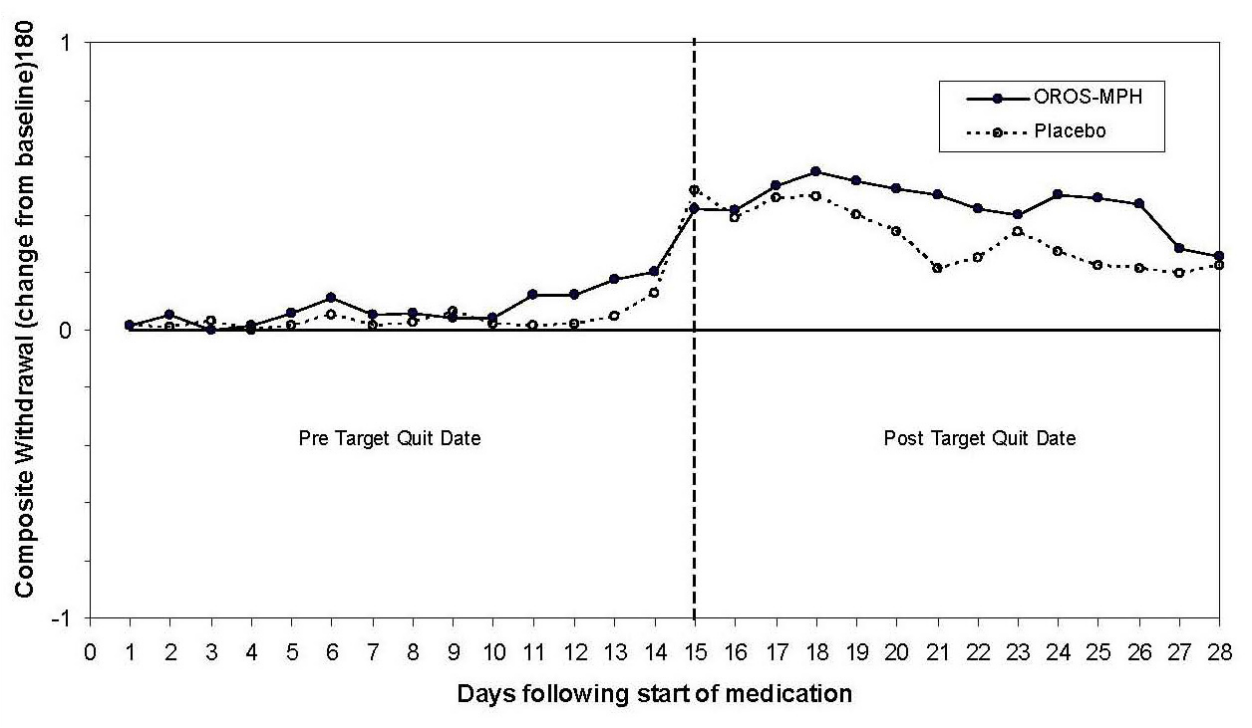
**Composite nicotine withdrawal scones before and after the target quit date**.

The composite nicotine withdrawal score change from baseline for the first 28 days following the start of medication was almost identical in the 2 groups. Nicotine withdrawal was not found to differ significantly between treatment groups during the first 14 days following the start of medication prior to the target quit date (p = 0.464) or during the first 14 days following the target quit date (p = 0.786). Groups also did not differ significantly for the individual item assessing craving (p = 0.724 and p = 0.350 for days 1-14 and 15-28 following the start of medication). Figure 1 graphically shows the composite nicotine withdrawal score as change from baseline through week four, which was two weeks following target quit date. Individual withdrawal symptoms were analyzed separately and the only significant finding was for restlessness, which was increased in the OROS-MPH subjects compared with placebo subjects (p = 0.01 and p = 0.067 from days 1-14 and 15-28 respectfully). This finding is consistent with the reported adverse events where restlessness was more frequent in the OROS-MPH group.

### Adverse Events

The percentage of subjects who reported one or more adverse events which were considered to be possibly, probably, or definitely related to study drug was significantly higher in those receiving OROS-MPH versus placebo (47.5% vs 15.0%; p = 0.003). Table 3 summarizes the reported adverse in the two groups. There were 5 subjects in the OROS-MPH group who dropped out of the study because of these adverse events: insomnia, insomnia and nausea, agitation, over-stimulation and substernal chest pain, all of which resolved with discontinuation of OROS-MPH.

**Table 3:** Adverse Events*

Event	Placebo N = 40	OROS-MPH N = 40
Restless	2 (5.0)	5 (12.5)
Insomnia	0 (0.0)	5 (12.5)
Headache	1 (2.5)	3 (7.5)
Anorexia	0 (0.0)	3 (7.5)
Vivid Dreams	1 (2.5)	2 (5.0)
Nausea	1 (2.5)	2 (5.0)
Anxiety	0 (0.0)	1 (2.5)
Chest Pain	0 (0.0)	1 (2.5)
Increased Thirst	1 (2.5)	0 (0.0)
Musculoskeletal Discomfort	0 (0.0)	1 (2.5)
Restless Leg	0 (0.0)	1 (2.5)
Weight Loss	0 (0.0)	1 (2.5)

* Adverse events considered to be possibly, probably, or definitely related to study drug are summarized according to treatment group. Overall, there were 6 (15%) subjects in the placebo group and 19 (47.5%) subjects in the methylphenidate group who reported one or more adverse events which were considered to be possibly, probably, or definitely related to study drug.

## Conclusions

In this pilot study of non-ADHD smokers, we evaluated the potential of OROS-MPH to help smokers to stop smoking, and observed no evidence of efficacy. Though based on the mechanism of action of OROS-MPH biologically plausible hypotheses exist that would support its potential usefulness as a treatment for tobacco dependence, we found no evidence to support such hypotheses. If a true therapeutic effect existed, we would have expected to see some signal. We found no evidence for any therapeutic effect either at the end of the medication phase or at the end of week 24. In addition, we saw no effect of OROS-MPH for nicotine withdrawal symptom relief and there was no reduction (or increase) in smoking rates observed in the OROSMPH group compared with the placebo group. Our results contrast with those in an open-label methylphenidate trial in 19 smokers [4]. In that study, participants received short-acting methylphenidate twice a day with a target dose of 20-30 mg/day and 12/19 (63%) reported withdrawal symptom relief with 5/19 reporting moderate relief. Our results are consistent with the larger study of smokers with ADHD where OROS-MPH did not increase smoking abstinence rates, but did improve ADHD symptoms [7].

One possible explanation for the lack of efficacy in our study is that the dose we used was not high enough to have an effect on the central nervous system dopamine levels in these smokers. The maximum dose we used was 54 mg/day while ADHD is routinely treated with doses twice that. However, in the study of smokers with ADHD the dose of OROS-MPH was titrated to 72 mg/d and there was no increase in smoking abstinence rates compared with the placebo group. It should be noted, however, that restlessness and insomnia were reported more often in those

taking OSOR-MPH, and it is likely those adverse event reports would increase if a higher dose was used. Given higher rates of adverse events reported in the OROS-MPH group, we do not believe using a higher dosage is warranted for a general population of smokers.

We did experience a substantial number of dropouts in this study which is similar to two recent studies of novel pharmacotherapy for treating tobacco dependence from the same research program [14,15]. Further, the placebo group had a 10% smoking abstinence rate, which is lower than the average placebo rate we typically observed in earlier years of our program, but may reflect that current smokers are more difficult to treat [8,16]. Consistent with our low placebo group rate, a recent multicenter study of varenicline in smokers with COPD found smoking abstinence rates in the placebo groups of less than 10% [17].

The strengths of this study include a prospective randomized placebo controlled design. We also used well developed behavioral interventions for smokers that we have employed effectively in many other clinical trials of pharmacotherapy for tobacco dependence. Further, we used a variety of standardized and validated instruments and procedures for participant assessments. Because we enrolled generally healthy smokers with no significant medical or psychiatric comorbid factions, our results would not likely be generalizable across the broad population of smokers. However, trial design and exclusions maximized our ability to see a signal supporting efficacy which was absent in this study. Our results do not support the use of OROS-MPH for the treatment of tobacco dependence.

## Competing interests

The authors declare that they have no competing interests.

## Authors' contributions

RDH developed the protocol and wrote the initial draft of the manuscript. JOE, AS, and JTH assisted in protocol writing, subject enrollment, study completion, and manuscript writing. ITC served as the study coordinator and oversaw all regulatory aspects of the study and data close-out procedures. DRS provided the statistical expertise during the protocol and manuscript writing. All authors read and approved the final manuscript.
